# Eridan de Medeiros Coutinho (★1931 †2024)

**DOI:** 10.1590/0037-8682-0166-2024

**Published:** 2024-06-10

**Authors:** Constança Simões Barbosa, Djalma Agripino de Melo, Sinval Pinto Brandão-Filho

**Affiliations:** 1 Fundação Oswaldo Cruz, Instituto Aggeu Magalhães, Departamento de Parasitologia, Recife, PE, Brasil.; 2 Universidade Federal de Pernambuco, Programa de Pós-Graduação em Saúde Coletiva, Centro de Ciências Médicas, Recife, PE, Brasil.; 3 Fundação Oswaldo Cruz, Instituto Aggeu Magalhães, Departamento de Imunologia, Recife, PE, Brasil.

On March 21st, at the age of 92, Professor Eridan de Medeiros Coutinho, a memorable teacher and researcher from Pernambuco, passed away.

While still a fifth year medical student at the University of Recife (currently the Federal University of Pernambuco (UFPE)), in 1953, Eridan began her brilliant research career. She started as an intern at the Pathology Laboratory of the Aggeu Magalhães Institute (IAM/Fiocruz), with the support of Professor Frederico Simões Barbosa, who, at that time, built this research unit of the Ministry of Health, opened in 1950, later linked to the Oswaldo Cruz Foundation in 1970. Eridan was born in Recife on August 7, 1931, the daughter of a father from Ceará and mother from Pernambuco. She was the mother of three children: Frederico Abath (*in memoriam*), a doctor and researcher at IAM/Fiocruz; Carlos Abath, an interventional radiologist and endovascular surgeon; and Ronaldo Abath, a lawyer and executive. Graduating in medicine from the UFPE in 1954, she chose, without hesitation, as she always remembered, to follow her vocation in pathology working exclusively in a career of research and teaching. As an academic assistant, she became a research technician after completing her medical degree. After completing her specialist training, she held the position of a medical epidemiologist. She completed a master’s degree in public health nutrition (1974) and a doctoral degree in nutritional pathology (1977), both from the UFPE, at a time when only men has such prominence.

**Figure:**
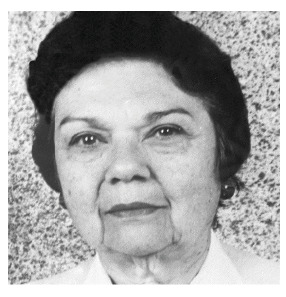

Her postgraduate training had already been developed previously, taking courses and internships at the University of São Paulo, in 1955, and then at Harvard University (Boston, USA), in 1957, where she interned as a Kellog Foundation fellow.

In addition to being a researcher at the IAM, she was a professor at the UFPE since the late 1960s, leading studies in malnutrition and its association with the morbidity of endemic infectious diseases, in the Department of Nutrition. She was the first woman to gain tenure (former professorship) in the field of medicine in Pernambuco, becoming full professor of Pathological Anatomy at the UFPE.

Researcher Emeritus at the Oswaldo Cruz Foundation, an honor received in 2004 for the relevance of her scientific contribution and services provided to the institution, Eridan worked prominently in pioneering studies in the pathology of tropical diseases, particularly studying aspects of host malnutrition in the pathology and host-parasite relationship of schistosomiasis and other parasitic diseases. On this topic, her works were pioneering, with several citations by national and international authors. In addition to her significant research contributions, she was a permanent professor for several years and supervised several students in postgraduate programs at the UFPE and Fiocruz, in addition to the postgraduate program in pathology at the Federal University of Bahia (UFBA), carried out in conjunction with the Gonçalo Muniz Institut of Fiocruz.

Due to her notable leadership in schistosomiasis studies, she coordinated the organizing committee and chaired the Fiocruz International Symposium on Schistosomiasis in Recife, held in 1997. As an activist for science and its strengthening in the Northeast region, she was an enthusiastic member of the Brazilian Society of Tropical Medicine (SBMT) and other scientific societies. In recognition of her significant role in tropical medicine, she was honorary president of the 45th SBMT Congress held in Recife in 2009.

Eridan also dedicated time in her extensive academic career to management positions at the IAM, as substitute head of IAM in the 1960s and 1970s, head of the Laboratory and then of the Department of Pathology. She was elected director of the IAM in 1993, and fulfilled her mandate until 1997 with total dedication and commitment to strengthening this oldest regional unit of Fiocruz. One of the main achievements during her administration was the Institute's first postgraduate program, the master’s degree in public health in 1996, in addition to improvements of facilities in the research area with renovations carried out in the laboratories and the construction of the block she named, with great satisfaction and enthusiasm, for Samuel Pessoa.

Throughout her career, she received more than 15 awards and honors, most notably, the Medal of Medical Merit (Academia Pernambucana de Medicina) in 2009, the Medal of Merit for Medical Education Professor Otávio de Freitas (Escola Pernambucana de Medicina) in 2008, the Pirajá da Silva Medal (Ministry of Health) in 2008, the Oswaldo Cruz Medal (Fiocruz) in 1995, the Pirajá da Silva Award in 1995, and the Carlos Chagas Prize in 1962.

Due to her enthusiasm for the history of the IAM, affectionately called Aggeu, Eridan was always present at moments celebrating the history of the institution and public health in the Northeast region, leading the preparation of the first book of its’ history entitled “Memories Revisited: The Aggeu Magalhães Institute in the lives of its characters” and published in 1997^1^, and as one of the organizers of the book of the institution’s 70 years, “Instituto Aggeu Magalhães: 70 years of Research and Teaching for Health”, published in 2020^2^, in addition to the events held in 2016 to celebrate the centenary of Professor Frederico Simões Barbosa.

Working relationships with Eridan as a colleague were guided by integrity and ethics while conducting research. She excelled in choosing a method that guaranteed faithful results and rigorously supervised data collection, and the results were exhaustively checked and analyzed; each article was a refined work led by the baton of a competent *Maestra*. At work meetings, she commanded the audience; she was not afraid of academic discussions, during which she imposed herself with perfect rhetoric and was infused with comprehensive knowledge. She was affectionately nicknamed *Generala*, by those closest to her, due to the firmness and objectivity with which she conducted meetings and decision-making.

For decades, Fred Abath, her beloved son and our late friend, carried out research in the same area and perhaps due to our long and intimate relationship with Eridan, we were the targets of many reprimands when we dared to escape her strict rules, but later everything could be laughed at, given new meaning by her witty and sharp humor. Throughout her long and brilliant career with a beautiful legacy, she will remain in the memory of everyone at the IAM, Fiocruz, and the scientific community in Pernambuco, the Northeast and Brazil.
